# Beta Cell 5′-Shifted isomiRs Are Candidate Regulatory Hubs in Type 2 Diabetes

**DOI:** 10.1371/journal.pone.0073240

**Published:** 2013-09-09

**Authors:** Jeanette Baran-Gale, Emily E. Fannin, C. Lisa Kurtz, Praveen Sethupathy

**Affiliations:** 1 Department of Genetics, University of North Carolina at Chapel Hill, Chapel Hill, North Carolina, United States of America; 2 Curriculum in Bioinformatics and Computational Biology, University of North Carolina at Chapel Hill, Chapel Hill, North Carolina, United States of America; 3 Lineberger Comprehensive Cancer Center, University of North Carolina at Chapel Hill, Chapel Hill, North Carolina, United States of America; University of Crete, Greece

## Abstract

Next-generation deep sequencing of small RNAs has unveiled the complexity of the microRNA (miRNA) transcriptome, which is in large part due to the diversity of miRNA sequence variants (“isomiRs”). Changes to a miRNA’s seed sequence (nucleotides 2–8), including shifted start positions, can redirect targeting to a dramatically different set of RNAs and alter biological function. We performed deep sequencing of small RNA from mouse insulinoma (MIN6) cells (widely used as a surrogate for the study of pancreatic beta cells) and developed a bioinformatic analysis pipeline to profile isomiR diversity. Additionally, we applied the pipeline to recently published small RNA-seq data from primary human beta cells and whole islets and compared the miRNA profiles with that of MIN6. We found that: (1) the miRNA expression profile in MIN6 cells is highly correlated with those of primary human beta cells and whole islets; (2) miRNA loci can generate multiple highly expressed isomiRs with different 5′-start positions (5′-isomiRs); (3) isomiRs with shifted start positions (5′-shifted isomiRs) are highly expressed, and can be as abundant as their unshifted counterparts (5′-reference miRNAs). Finally, we identified 10 beta cell miRNA families as candidate regulatory hubs in a type 2 diabetes (T2D) gene network. The most significant candidate hub was miR-29, which we demonstrated regulates the mRNA levels of several genes critical to beta cell function and implicated in T2D. Three of the candidate miRNA hubs were novel 5′-shifted isomiRs: miR-375+1, miR-375-1 and miR-183-5p+1. We showed by *in silico* target prediction and *in vitro* transfection studies that both miR-375+1 and miR-375-1 are likely to target an overlapping, but distinct suite of beta cell genes compared to canonical miR-375. In summary, this study characterizes the isomiR profile in beta cells for the first time, and also highlights the potential functional relevance of 5′-shifted isomiRs to T2D.

## Introduction

miRNAs are short regulatory RNAs that are processed from variable length primary transcripts through consecutive ribonuclease-mediated cleavage events [1], [2]. miRNAs guide and tether the RNA induced silencing complex (RISC) to specific RNAs in order to regulate their stability and/or translation [3]. Numerous studies have identified miRNAs as important modulators of a wide variety of biological pathways [4], [5]; for example, miR-375-mediated gene regulation is critical for both beta cell development and function [6], [7].

**Figure 1:**
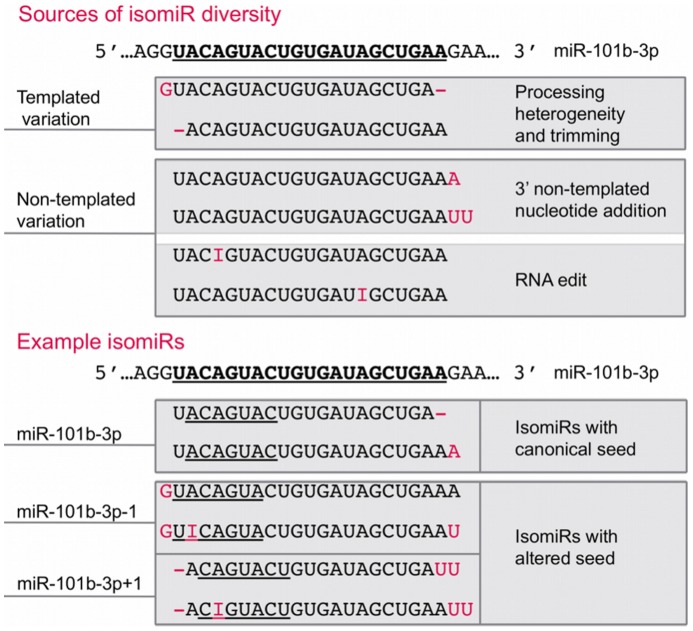
Sources of isomiR diversity. The top panel illustrates sources of isomiR diversity, which stratify into two classes: templated and non-templated variations. As illustrated by the bottom panel, an isomiR may contain one or both types of variations, and both templated (e.g. 5′-shifts) and non-templated (e.g. RNA edits) variations can create an isomiR with an altered seed. The seed region of each isomiR is underlined.

Similar to protein coding genes, miRNAs are present in multiple isoforms, called isomiRs [8]–[10]. IsomiRs are sequence variants, generated from a single miRNA locus, that consist of one or both of two types of variations: templated and non-templated [9], [11], [12] (Fig. 1). Templated variants match the genomic sequence, but have differing 5′-start and/or 3′-end positions, likely due to processing heterogeneity by Drosha/Dicer [1], [2] and/or exonuclease-mediated nucleotide trimming [13], [14]. Non-templated isomiRs are diverged from the genomic sequence due to post-transcriptional enzymatic processes that add, remove, or edit specific nucleotides [10]. Nucleotide additions are catalyzed by a class of enzymes called ribonucleotidyl transferases, which modify miRNAs by covalent addition of nucleotides to the 3′-end [15]. The most prevalent form of RNA editing is the adenosine-to-inosine edit, which is mediated by the double-stranded RNA adenosine deaminase (ADAR) family of enzymes [16].

IsomiRs were initially dismissed as byproducts of technical (e.g. sequencing errors) or biological noise [17], [18]. However, recent studies have shown that isomiRs interact with the RISC and are present in polysomes [19]–[22], suggesting that they may be biologically relevant. Several studies have demonstrated that 3′-non-templated nucleotide additions (3′-NTAs), most commonly uridylation or adenylation [19], [22]–[27], affect miRNA stability and/or loading onto the RISC [10], [22], [23] and are physiologically regulated [15], [28]. Also, several studies have identified isomiRs generated by RNA edits at the 5′-end of the miRNA [29]–[32], referred to as the seed region, which is a critical determinant of stable miRNA targeting [3]. Modifications to the seed region have the potential to redirect a miRNA to a vastly different set of target RNAs, thereby potentially altering its biological function [29]. Perhaps the best-studied example of this phenomenon is the A-to-I editing of the miR-376 primary transcript leading to the expression of a 5′-isomiR of miR-376 with a modified seed [29]. The canonical version of miR-376 and its seed-altered isomiR were shown to have highly distinct target sets [29], highlighting the biological importance of 5′-isomiRs.

5′-isomiRs are not limited to those generated by RNA edits; they can also be produced by processing heterogeneity and/or 5′-end nucleotide trimming, which can shift the 5′-start positions of miRNAs (Fig. 1). 5′-shifted isomiRs have been identified in a few recent studies [19], [22], [24], [33]–[35]; however, they are often reported to be lowly expressed [19], [24] and continue to be perceived as rare [9]. As such, they are often overlooked by deep sequencing studies, including those performed in pancreatic beta cells. Because the 5′-end of a miRNA is so critical for function, it is of substantial interest to characterize comprehensively the prevalence and physiological relevance of miRNA 5′-diversity.

To that end, we developed an in-house bioinformatic analysis pipeline for the quantitation of miRNAs and their 5′-isomiRs in mouse insulinoma cells (MIN6), which are widely used as a surrogate for pancreatic beta cells [36]. Further, we applied the pipeline to published small RNA-seq datasets from primary human beta cells and whole islets. Strikingly, we found not only that the miRNA expression profile in MIN6 cells correlates very well with those of the primary human beta cells and islets (r^2^>0.98), but also that 7 highly expressed 5′-shifted isomiRs in MIN6 cells are also abundant in human beta cells and whole islet. Finally, using a Monte Carlo simulation strategy, we identified ten beta cell miRNAs, including three 5′-shifted isomiRs, as significant candidate regulatory hubs in a T2D gene network.

## Materials and Methods

### Small RNA-seq Datasets

#### Small RNA-seq

MIN6 cells were cultured in high glucose (25 mM) DMEM (Sigma) supplemented with 10% heat-inactivated fetal bovine serum. Cells were lysed and total RNA was extracted using the Norgen total RNA purification kit. RNA quality was assessed by Agilent 2100 Bioanalyzer, and only very high quality samples with RNA Integrity Number (RIN) above 9.0 were considered further. Small RNA libraries (three biological replicates) were generated using the Illumina TruSeq small RNA library preparation kit. These libraries were then sequenced on the Illumina HiSeq platform. Small RNA-seq data are available in the GEO database (Accession ID: GSE44262). Sequencing of small RNAs from mouse liver was conducted as well, in accordance with the protocol described above.

#### Human primary cell data

Primary beta cell and whole islet small RNA-seq datasets were obtained from GEO (GSE47720: [37]). This study included two libraries of beta cells (GSM1155397 and GSM1155398) and one whole islet sample (GSM1155395) that were prepared with the Illumina TruSeq protocol and sequenced on the Illumina HiSeq platform.

### Small RNA-seq Read Mapping Pipeline

Small RNA-seq reads are first trimmed by cutAdapt (parameters -O 10–e 0.1) to remove remnants of the 3′-adaptor sequence [38]. Next, trimmed reads of size 14–41 nt are mapped, without mismatches allowed, to the reference genome using Bowtie [39] (parameter set: -q -a -m 20 -n 0 -e 70). All mapped reads separated by 65 nt or less are merged. These merged windows are then extended by 5 nt on either end. All remaining reads (those that could not be mapped exactly by Bowtie) are aligned with mismatches allowed to the extended windows using SHRiMP2 [40].

Specifically, the set of all possible “alignment seeds” containing one mismatch in the body (M_0_) and up to three mismatches at the 3′-end (depending on read length) is generated and used to align all reads to the genomic windows. The number of mismatches allowed at the 3′-end (M_1_) for a read of length L is defined as: M_1_ = 0 if L<16, M_1_ = 1 if 16≤L<19, M_1_ = 2 if 19≤L≤23, and M_1_ = 3 if L>23. Finally, all reads mapping equally well to multiple loci are proportionally assigned to those loci. Mapped reads are grouped by 5′-start position (5′-isomiRs) and are annotated with respect to the start position of the reference miRNA (miRBase r18) at the same locus [41]. For each miRNA or 5′-isomiR, all reads with mismatches at the 3′-end are counted as 3′-NTAs. Mismatches within a read are counted as potential RNA edits. For additional details see supplemental methods (File S1).

### Candidate miRNA Regulatory Hub Identification in the T2D Gene Network

#### T2D gene list

We identified the nearest genes to each genetic variant significantly associated with T2D (p-values <10^−7^) from (1) the T2D genome-wide association studies (GWAS) listed in the NHGRI catalog (http://www.genome.gov/gwastudies) and (2) a T2D GWAS reported by Morris *et al*. that was not included in the NHGRI catalog [42]. Additionally, we included twenty-three genes linked to maturity onset diabetes of the young (MODY), neonatal diabetes (NDM), and chronic hyperinsulinemia (CHI). The total number of genes was 92.

#### Identification of miRNA regulatory hubs

Candidate miRNA regulatory hubs in the T2D gene network were identified by Monte Carlo simulation analysis. First, we used the seed-based target prediction algorithm TargetScanS 5.2 [43] to determine for each beta cell miRNA the number of predicted conserved targets among the human genes in the T2D network. Each predicted miRNA – gene interaction was assigned a score based on the strength of the seed match, the level of conservation of the target site, and the clustering of target sites within that gene’s 3′-UTR. Additionally, the score for each gene was weighted according to the number of high-confidence protein-protein interactions reported in the STRING 9.0 database [44]. Finally, for each miRNA, the final targeting score was calculated by summing the scores across all genes and dividing by the number of genes. We repeated this procedure 30,000 times, with a new set of randomly selected human genes each time, in order to generate a background distribution of the predicted targeting scores for each miRNA (genes and corresponding 3′-UTR sequences were downloaded from http://www.targetscan.org). These score distributions were then used to calculate an empirical p-value of the targeting score for each miRNA in the T2D gene set. Genes were selected at random from a pool with similar overall connectivity to the genes in the T2D gene set, and to account for differences in the average 3′ UTR length between the genes of interest and the randomly selected genes in each simulation, the targeting score was normalized by 3′ UTR length. For additional details see supplemental methods (File S1).

### Validation of miRNA-mediated Gene Regulation

MIN6 cells were transiently transfected with (1) 10 nM mmu-miR-29 mimic (Dharmacon); (2) 200 nM mmu-miR-29 hairpin-inhibitor (Dharmacon); (3) 10 nM mmu-miR-375 mimic (Dharmacon); (4) 10 nM custom mmu-miR-375+1 mimic (Dharmacon: 5′-UUGUUCGUUCGGCUCGCGUGA-3′) or (5) 10 nM custom mmu-miR-375-1 mimic (Dharmacon: 5′UUUUGUUCGUUCGGCUCGCGUGA-3′). After 48 hours, RNA was extracted from cells using the Norgen Total RNA Purification Kit, and miRNA and mRNA levels were measured by real-time quantitative PCR (RT-qPCR) using TaqMan microRNA and gene assays (Applied Biosystems).

## Results

### Comparing the miRNA and 5′-isomiR Profiles of MIN6, Human Beta Cell, and Human Islet

To characterize isomiR diversity (Fig. 1) in mouse insulinoma cells (MIN6), we generated small RNA libraries (n = 3) and performed deep sequencing on the Illumina HiSeq platform (Methods), which yielded ∼18 million reads per replicate. After 3′-adaptor trimming, on average ∼50% of the reads were within the expected size range (16–27 nt) for miRNAs (Table S1 in File S2). To analyze the small RNA-seq reads further, we developed and implemented an in-house bioinformatic analysis pipeline for highly sensitive detection and quantitation of isomiRs (Methods, Fig. S1 in File S1). We successfully mapped ∼92% of the trimmed MIN6 reads in each replicate and on average ∼75% of the mapped reads corresponded to annotated miRNA loci (Table S1 in File S2).

We also used our pipeline to analyze published small RNA-seq datasets from primary human beta cells (n = 2) and whole islet (n = 1) [37]. These datasets had approximately 41, 33 and 79 million reads respectively, and in each case more than 80% of the 3′-adaptor trimmed reads were within the expected size range (16–27 nt) for miRNAs (Table S1 in File S2). We successfully mapped ∼97% of the trimmed reads and on average ∼85% of the mapped reads corresponded to annotated miRNA loci (Table S1 in File S2).

In each of the datasets, >1,000 distinct mature miRNAs were represented by at least ten reads. However, 98% of the miRNA-related reads captured the top ∼100–150 mature miRNAs depending on the dataset. We refer to these miRNAs as “highly expressed.” To compare miRNA profiles across the MIN6 replicates and human samples, we first assembled a list of miRNAs that were “highly expressed” in at least one dataset, resulting in 209 distinct mature miRNAs produced from 187 unique pre-miRNAs (Table S2 in File S2). These 187 pre-miRNAs consisted of:

1. 166 pre-miRNAs that generate at most one mature miRNA from each arm of the hairpin-like structure (“homogenous loci”), including one locus (pre-miR-5099) that produces only a 5′-shifted isomiR (mmu-miR-5099-2); and
2. 21 pre-miRNAs that generate more than one mature miRNA from the same arm (“heterogeneous loci”), including one locus (pre-miR-375) that produces one 5′-reference miRNA and two 5′-shifted isomiRs.

**Figure 2:**
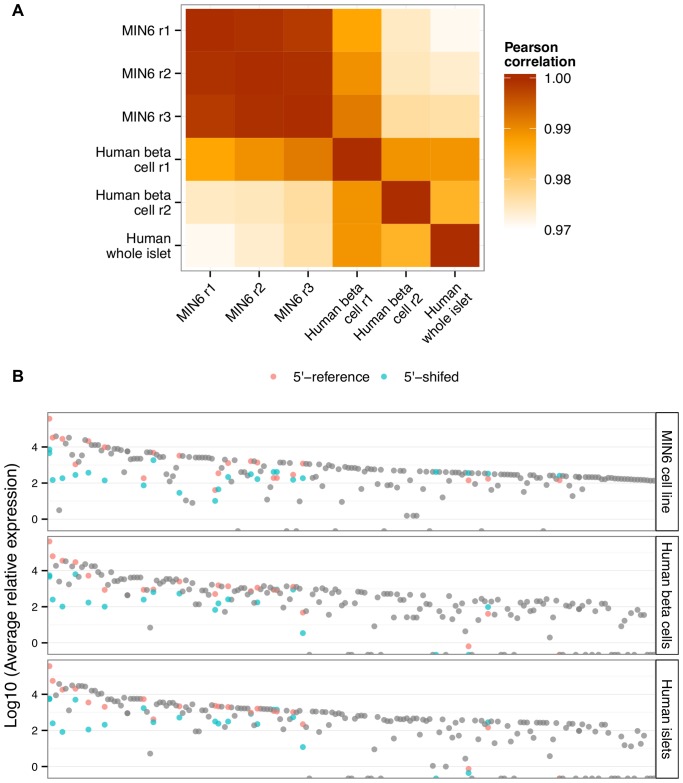
miRNA and isomiR profiles in MIN6 cells, primary human beta cells and human islet. (**A**) A heatmap is shown depicting the Pearson correlation coefficients of miRNA profiles between pairs of samples analyzed in this study. (**B**) The x-axis depicts highly expressed miRNAs ordered from left to right by decreasing maximal expression across all samples. The y-axis depicts the Log10 of the average read count per million. Each dot represents a miRNA. miRNAs from a homogenous locus (a pre-miRNA that produces only one mature miRNA per arm of the hairpin) are in gray. miRNAs from a heterogeneous locus (a pre-miRNA that produces more than one mature miRNA per arm of the hairpin) are either pink (5′-reference) or blue (5′-shifted).

Among the 209 mature miRNAs, 186 were 5′-reference miRNAs and 23 were 5′-shifted isomiRs (Table S2 in File S2). The miRNA profiles of each of the MIN6 replicates correlated extremely well with each other (r^2^∼0.99) and, strikingly, also with those of the human beta cells (average r^2^ = 0.98) and whole islets (average r^2^ = 0.97) (Fig. 2A; Table S2 in File S2). As a control, we also sequenced libraries of small RNAs from the mouse liver prepared according to the same protocol and determined that as expected the correlation with the MIN6 profile was very poor (r^2^<0.1; data not shown). The isomiRs from the heterogeneous loci were distributed across the spectrum of highly expressed miRNAs (Fig. 2B), indicating that the heterogeneous status of a miRNA locus is not merely a function of the level of expression.

**Figure 3:**
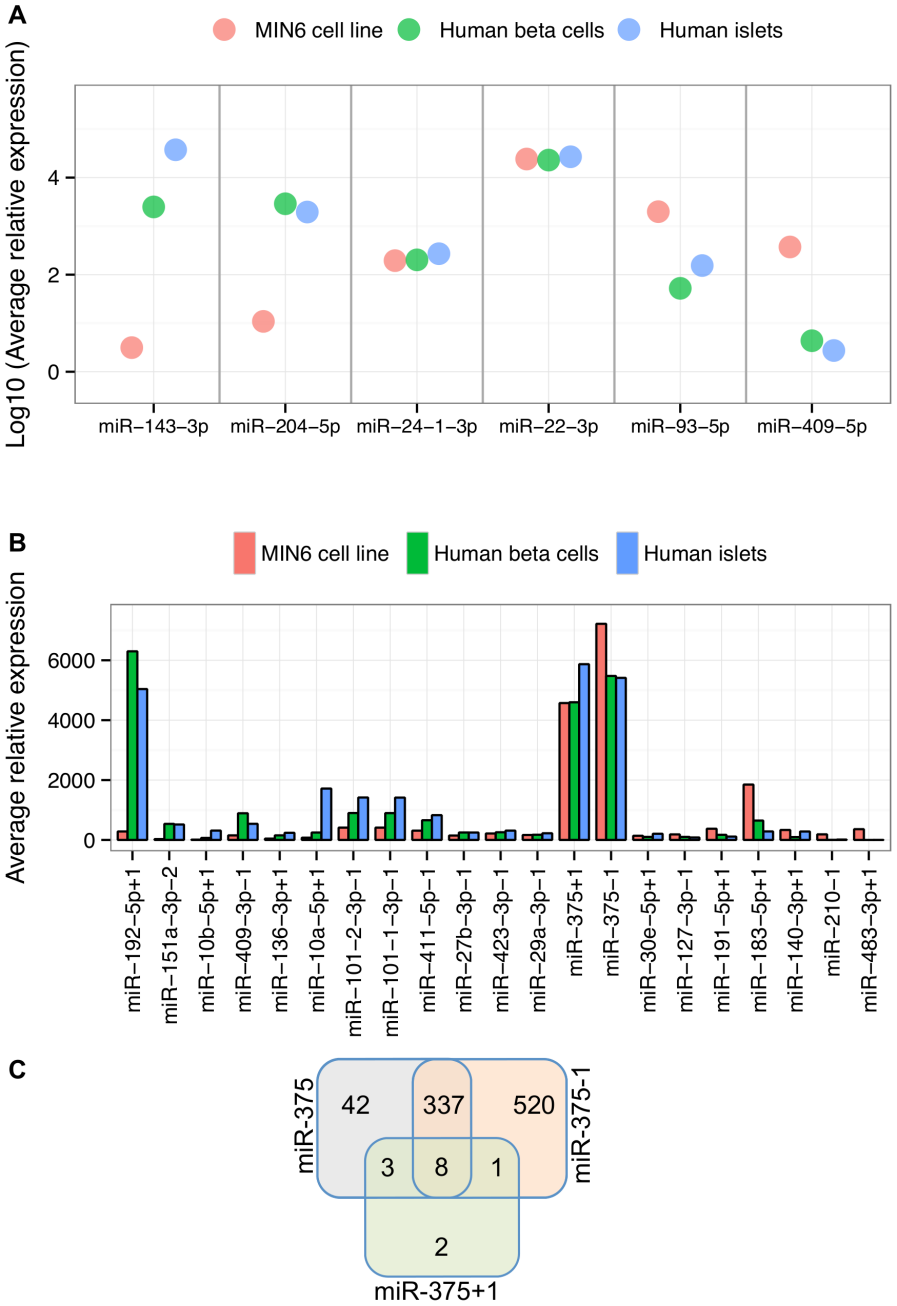
Comparison of 5′-reference miRNA and 5′-shifted isomiR expression levels among MIN6 cells, human beta cells, and human islet. (**A**) The x-axis lists selected 5′-reference miRNAs in MIN6 (red), human beta cells (green), and human islets (blue). The y-axis depicts the Log10 of the average read count per million for each 5′-reference miRNA in each sample. (**B**) The x-axis shows the highly expressed 5′-shifted isomiRs ordered from left to right by decreasing fold-difference between primary human beta cells and MIN6 cells. The y-axis depicts the average read count per million for each 5′-shifted isomiR. (**C**) The number of genes with at least one conserved target site for miR-375 (gray), miR-375+1 (green), and miR-375-1 (orange) is shown. All sets are mutually exclusive: for example, a total of 390 genes have predicted conserved miR-375 target sites (42 unique to miR-375, 3 shared with miR-375+1 only, 337 shared with miR-375-1 only, and 8 common to all three).

Although miRNA expression among these samples was highly correlated overall, such as in the case of miR-22-3p or miR-24-1-3p (Fig. 3A), several miRNAs appeared to be specifically or preferentially expressed in either the MIN6 cells or human beta cells/islets (Fig. 3A). For example, miR-143-3p and miR-204-5p were 791- and 265-fold more highly expressed, respectively, in the human beta cells than in the MIN6 cell line (Fig. 3A). Likewise, miR-93-5p and miR-409-5p were 38- and 85-fold more highly expressed, respectively, in MIN6 cells than in human beta cells (Fig. 3A).

### Characterization of Beta Cell 5′-shifted isomiRs

Of the 23 highly expressed MIN6 5′-shifted isomiRs, only mmu-miR-5099-2 and mmu-miR-101b-3p-1 did not have a homologous miRNA in the human samples. Among the remaining 21, two were in the set of top 20 most highly expressed miRNAs in each of the MIN6 and human datasets: miR-375+1 and miR-375-1. Many of the 5′-shifted isomiRs, such as miR-375+1, miR-375-1, and miR-27b-3p-1, were expressed at similar levels in MIN6, human beta cell, and human islet samples (Fig. 3B). However, some 5′-shifted isomiRs were preferentially associated with either MIN6 or one of the human samples. For example, miR-192-5p+1 was 22-fold more highly expressed in human beta cells than in MIN6, miR-10a-5p+1 was 23-fold more highly expressed in human islets than in MIN6, and miR-183-5p+1 was nearly 3-fold more highly expressed in MIN6 than in human beta cells or islets (Fig. 3B). These differences are likely in part a reflection of the disparities in cellular composition among MIN6 cells, beta cells, and whole islets. Nevertheless, the overall profile of 5′-shifted isomiRs was fairly highly correlated between MIN6 and human beta cells/islets (r^2^∼0.7).

To illustrate the potential regulatory impact of these 5′-shifted isomiRs we used TargetScan [43] to predict targets for miR-375 and its 5′-shifted isomiRs, miR-375+1 and miR-375-1. While miR-375 has 390 predicted targets conserved between human and mouse, miR-375-1 targets has more than twice that many, and strikingly, miR-375+1 has only 14 (Fig. 3C). Only eight genes (*ELAVL4, HNF1B, NFIX, NPAS3, PAX2, SHOX2, SLC16A2,* and *TSC22D2*) have predicted conserved target sites for miR-375 and both of its 5′-shifted isomiRs.

### Candidate 5′-shifted isomiR Regulatory Hubs in Type 2 Diabetes

**Figure 4:**
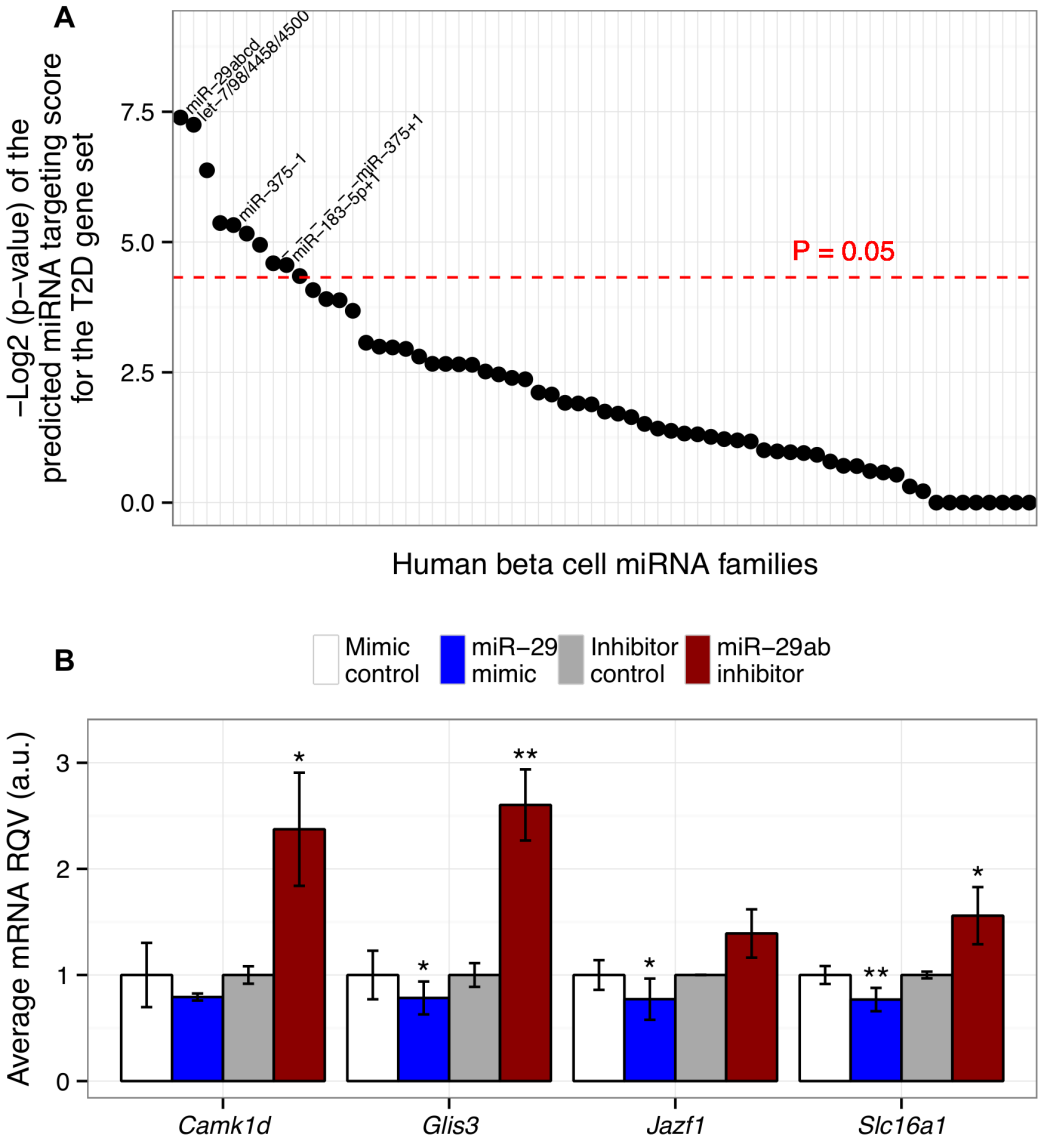
Candidate miRNA regulatory hubs in a type 2 diabetes gene network. (**A**) Each data point represents a 5′-reference miRNA or a 5′-shifted isomiR from primary human beta cells, and the y-axis shows the negative Log2 of the p-value of the predicted miRNA targeting score among genes in a type 2 diabetes (T2D) network. The dashed red line denotes the significance threshold (empirical P = 0.05). (**B**) Effects of miR-29 mimic and inhibitor in MIN6 cells on the mRNA levels of four T2D genes are shown. The x-axis lists the gene symbols for each of four predicted miR-29 target genes and the y-axis depicts the relative quantitative value (RQV; expression determined by RT-qPCR and normalized to *Rps9*) in response to the miR-29 mimic (blue) or the miR-29 inhibitor (red) relative to mock transfection. The data shown represent at least two independent experiments, each conducted in triplicate. P-values were calculated based on Student’s t-tests. *, P<0.05; **, P<0.01.

Genome-wide association studies for type 2 diabetes (T2D) have primarily (though not exclusively) implicated genes with critical function in the pancreatic beta cell [45], [46]. Therefore, we sought to determine if any of the highly expressed human beta cell miRNAs, including 5′-shifted isomiRs, serve as regulatory hubs in T2D. We first assembled a list of genes (n = 92) implicated in T2D and related conditions including maturing onset diabetes of the young (MODY) (Methods). We then implemented a Monte Carlo simulation strategy (Methods) to determine for each miRNA whether the predicted regulatory impact on T2D genes is significantly (uncorrected P<0.05) greater than expected by chance (such miRNAs are termed “candidate regulatory hubs”). We identified 10 candidate miRNA regulatory hubs (Fig. 4A; Table S3 in File S2). The top two were the 5′-reference miRNAs miR-29 and let-7, both of which have been implicated in beta cell function and glucose homeostasis [47]–[49]. Though miR-29 has been shown to regulate glucose-stimulated insulin secretion, its target genes in the beta cell are largely unknown. To validate the *in silico* approach, we selected several predicted targets (*Camk1d*, *Glis3*, and *Jazf1*), and one previously validated target (*Slc16a1* [48]), of miR-29 from among the T2D gene list for evaluation in MIN6 cells. Specifically, we transiently transfected MIN6 cells with a miR-29 mimic or inhibitor (antagomiR) and measured the mRNA levels of each of the four genes by real-time quantitative PCR (RT-qPCR). Three of the four genes were significantly (p<0.05) down regulated by the over-expression of miR-29 and three genes were significantly (p<0.05) up regulated by the antagomiR-mediated inhibition of miR-29 (Fig. 4B). These findings are consistent with previous reports that miR-29 is involved in the regulation of beta cell function [48], [50], and they serve as a validation of the *in silico* regulatory hub analysis.

Strikingly, three of the 10 candidate miRNA regulatory hubs in the T2D gene network were 5′-shifted isomiRs: miR-375+1, miR-375-1, and miR-183-5p+1 (Fig. 4A). Moreover, all three of these were more significantly associated with T2D genes than their 5′-reference counterparts (Table S3 in File S2). This is particularly intriguing, given the already well-established role of 5′-reference miR-375 in beta cell formation and function.

### 5′-shifted isomiRs of the Beta Cell-enriched miRNA, miR-375

**Figure 5:**
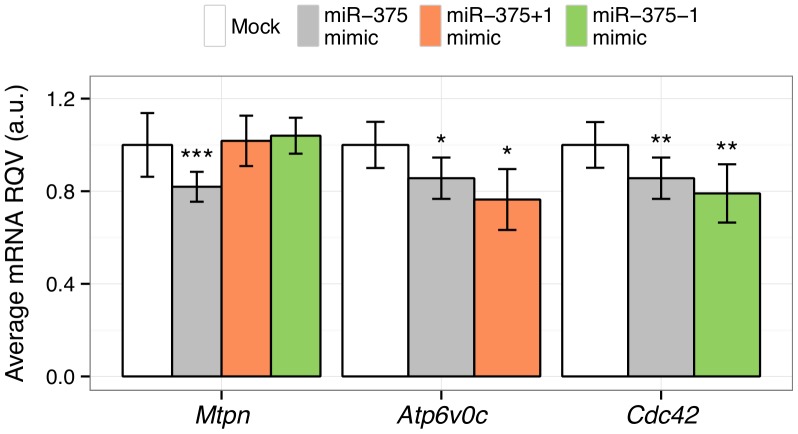
Evaluation of miR-375 and its 5′-shifted isomiRs in MIN6 cells. Effects of mimics for 5′-reference miR-375, 5′-shifted miR-375+1, and 5′shifted miR-375-1 in MIN6 cells on the mRNA levels of three genes are shown. *Mtpn* is a known target of 5′-reference miR-375 but not predicted as a target for either of the 5′-shifted miR-375 isomiRs; *Atp6v0c* is predicted to be preferentially targeted by miR-375+1; and *Cdc42* is predicted to be preferentially targeted by miR-375-1. The x-axis lists the gene symbols for each of three genes tested. The y-axis depicts the relative quantitative value (RQV; expression determined by RT-qPCR and normalized to *Rps9*) in response to the miR-375 mimic (gray), miR-375+1 mimic (orange), or miR-375-1 mimic (green) relative to mock transfection. The data shown represent at least two independent experiments, each conducted in triplicate. P-values were calculated based on Student’s t-tests. *, P<0.05; **, P<0.01, ***, P<0.001.

As depicted in Fig. 3C, miR-375 and its 5′-isomiRs have overlapping, but distinct predicted target gene profiles. To further evaluate the putative differential targeting of the miR-375 5′-isomiRs, we selected the following three genes: *Mtpn*, which regulates insulin secretion, is a known target of the 5′-reference miR-375 [6], but is not predicted to be targeted by the 5′-shifted isoforms; *Atp6v0c*, which mediates glucose-sensitive intracellular vesicular transport and is predicted to be preferentially targeted by the 5′-shifted isoform miR-375+1; and *Cdc42*, which is essential for the second phase of insulin secretion and is predicted to be preferentially targeted by the 5′-shifted isoform miR-375-1. We transfected MIN6 cells with (1) transfection reagent only (mock), (2) 10 nM of miR-375 mimic, or (3) 10 nM of a mimic for one of the 5′-shifted isomiRs of miR-375, and measured the mRNA levels of each of the three genes by RT-qPCR. *Mtpn* was repressed only by the 5′-reference miRNA (Fig. 5). *Atp6v0c* and *Cdc42* were also modestly repressed by the 5′-reference miRNA, though slightly more so by miR-375+1 and miR-375-1, respectively (Fig. 5). In each case, the strongest repression was conferred by the 5′-isomiR with the strongest predicted target site.

## Discussion

In this study we developed an in-house bioinformatic analysis pipeline to characterize isomiR diversity, and applied this method to study isomiR expression in the MIN6 cell line, primary human beta cells and islets. We found that (1) the miRNA expression profile in the MIN6 cell line is highly correlated with that of the primary human beta cells, (2) miRNA loci can be classified as either homogeneous (producing a single highly expressed 5′-isomiR) or heterogeneous (producing multiple highly expressed 5′-isomiRs), (3) 5′-shifted isomiRs can be as abundant as their 5′-reference counterparts, and (4) there are seven 5′-shifted isomiRs highly expressed in MIN6 cells that are also abundant in human beta cells and islets. Additionally, we identified 10 beta cell miRNAs, including three 5′-shifted isomiRs, as candidate regulatory hubs in type 2 diabetes. We evaluated several predicted gene targets of our top candidate regulatory hub, miR-29, and demonstrated the potential of the 5′-shifted isomiRs miR-375+1 and miR-375-1 to differentially regulate gene expression in MIN6 cells.

While the unambiguous validation of the targeting activity of 5′-shifted isomiRs is important, it is hindered by inherent limitations of the currently available technologies. For example, the gold standard experiment would be to specifically knock-down the 5′-shifted isomiR of interest. However, current strategies for knock-down (e.g. locked nucleic acids), and for testing the efficacy of the knock-down (e.g. TaqMan RT-qPCR), do not adequately distinguish between the 5′-reference and 5′-shifted isoforms. New approaches for studying miRNA function must be developed in order to tackle the technical challenges posed by 5′-shifted isomiRs, which are often identical in sequence to the 5′-reference form except for the addition/loss of a single nucleotide at the 5′-end. Though outside the scope of this study, further analyses are necessary to firmly establish the functional relevance of the 5′-shifted isomiRs. Nonetheless, to our knowledge, this is the first report of highly expressed 5′-shifted isomiRs in beta cells, several of which are candidate regulatory hubs in T2D.

The findings in this study promote the notion that 5′-shifted isomiRs are prevalent and potentially impact disease, thus widening the panoramic view of the functional miRNA-ome. In addition, we have identified for the first time three 5′-shifted isomiRs as significant candidate regulatory hubs in a disease network. The novel strategy employed in this study can be utilized for additional disease models to uncover potential roles for 5′-shifted isomiRs in the regulatory networks of complex diseases.

## Supporting Information

File S1 **Supporting methods and figures. Figure S1. Overview of tiered mapping strategy.** Trimmed reads are mapped in a tiered fashion to the genome. First, reads that map exactly to transcriptionally active regions (as determined by nascent RNA-seq) of the genome are used to create genomic windows. Next, any remaining un-mapped reads are allowed to map imperfectly only to the genomic windows generated in step 1, as opposed to the entire genome, thereby drastically reducing the mapable space. All reads that map equally well to multiple loci are proportionally assigned to all those loci.
(DOCX)

File S2 **Supporting tables. Table S1**: Table listing statistics for each of the small RNA-seq datasets used in this study. Biological replicates for MIN6 are annotated as MIN-1, MIN6-2, and MIN6-3. **Table S2**: This table lists the top miRNAs that constitute 98% of all reads mapping to miRNA loci (“highly expressed”). Each entry lists the mouse miRNA name and the corresponding human miRNA name (based on miRBase r18); if the miRNA is not found (meaning not present in miRBase r18) in either species it is noted with the prefix NF and the entry is highlighted in red. 5′-shifted isomiRs are highlighted in green. Each entry of the table lists the relative expression, expression rank, and % NTAs for each sample. MIN6 replicates are denoted by increasing sample number; primary human beta cell and whole islets are labeled by their SRA number. The relative expression of each miRNA is computed as the number of reads mapping to that miRNA over the total number of mapped reads multiplied by one million. As such, this number reports the expected relative expression per million mapped reads. The rank column reports the miRNA’s expression rank in each sample (a blank in the rank column indicates that that miRNA was not deemed to be “highly expressed” in that sample). The %NTA column indicates the percent of reads mapping to a particular miRNA reported to have a 3′-non-templted nucleotide addition. Additionally the average and standard deviation of expression for the MIN6 samples are provided in the dark blue column, and an average is provided for the 2 human beta cell libraries. **Table S3**: This table lists the predicted targets among T2D genes (n = 92) for miRNA families highly expressed in primary human beta cells. All target sites are required to be conserved among humans and two other species among mouse, rat, dog and chicken. Targeting score is calculated as detailed in the supplemental methods and miRNA families are sorted by empirical p-value.
(XLSX)
