# Supplementary material for: Beta Cell 5′-Shifted isomiRs Are Candidate Regulatory Hubs in Type 2 Diabetes
Source: PLoS One. 2013 Sep 9;8(9):e73240. doi: 10.1371/journal.pone.0073240 (PMC3767796; doi:10.1371/journal.pone.0073240)
Supplement: File S1 — Supporting methods and figures. Figure S1. Overview of tiered mapping strategy. Trimmed reads are mapped in a tiered fashion to the genome. First, reads that map exactly to transcriptionally active regions (as determined by nascent RNA-seq) of the genome are used to create genomic windows. Next, any remaining un-mapped reads are allowed to map imperfectly only to the genomic windows generated in step 1, as opposed to the entire genome, thereby drastically reducing the mapable space. All reads that map equally well to multiple loci are proportionally assigned to all those loci. (DOCX) [file pone.0073240.s001.docx]

# Supplemental Methods and Figures

**Small RNA-seq mapping pipeline:**

This pipeline is intended to process and quantify small RNA-seq reads mapping to (1) established miRNAs, and (2) potential novel miRNAs. miRNAs are small non-coding RNAs that are involved in the post-transcriptional regulation of mRNA. The 5’-end of miRNAs contains the “seed” region, which plays a significant role in directing miRNAs to their targets. miRNAs derived from the same pre-miRNA, having different 5’-start locations are called 5’-isomiRs. To enable us to capture the 5’-diversity of expressed isomiRs, reads are grouped by 5’-start and annotated by their offset relative to their miRBase (r18) designation.

**Figure S1. Overview of tiered mapping strategy.** Trimmed reads are mapped in a tiered fashion to the genome. First, reads that map exactly to transcriptionally active regions (as determined by nascent RNA-seq) of the genome are used to create genomic windows. Next, any remaining un-mapped reads are allowed to map imperfectly only to the genomic windows generated in step 1, as opposed to the entire genome, thereby drastically reducing the mapable space. All reads that map equally well to multiple loci are proportionally assigned to all those loci.

**Stage 1**: Generate trimmed reads file, and split into separate files based on read length.

*Input:* FASTQ file containing raw reads

*Parameters:*

1. Adapter overlap: number of bases of the 3’ adapter that must overlap with the read.
2. The allowed number of errors in the minimum length adapter. Preferred parameters: Overlap = 10, number of errors = 1.

*Output:*

1. Trimmed FASTQ file. Name format: rawName_O*x*_E*y*.fq where *x*= the minimum adapter overlap requested, and *y*= the number of errors allowed in the adapter sequence.
2. A separate file for each read length is generated with the name format: rawName_O*x*_E*y*_*z*.fq, where *x*= the minimum adapter overlap requested, *y*= the number of errors allowed in the adapter sequence, and *z* is the length of the reads stored in the file.

*Required tools:* The adapter trimming is performed with cutAdapt v1.0, and the reads are split into separate files based on read length using a custom Perl script.

*Motivation:* This stage of the pipeline prepares the raw reads for alignment to a reference genome. As small RNAs are often shorter than the length of a sequenced read, the remnants of the 3’-adaptor sequence must be removed prior to alignment.

**Stage 2**: Align trimmed reads to a reference genome (No mismatches).

*Input:* Size separated trimmed reads (rawName_O*x*_E*y*_*z*.fq output from stage 1).

*Parameters:*

1. Genome [hsa, mmu]
2. Read quality offset (33/64): this is the offset added to the phred score in the FASTQ quality scores. i.e. Sanger format is phred + 33 (Non-selectable) bowtie options: -q -a -m 20 -n 0 -e 70

*Output:*

1. fileName.hits: this file contains all alignments to the requested genome.
2. fileName.noHits: this file contains read that had no exact matches to the requested genome.

*Required tools:*  Bowtie 0.12.7

*Motivation:* This stage of the pipeline serves two purposes: (1) we acquire a best match for every read to the reference genome and (2) we interpret the regions surrounding these exact matches as transcriptionally active. The regions defined by these perfectly aligned reads are used to generate a set of genomic windows in which further alignments will be attempted.

**Stage 3**: Generate library of windows surrounding exact matches

*Input:*

1. The main input to this stage is the file containing the perfectly aligned reads: fileName.hits (output of stage 2).
2. Genome: [hsa, mmu] the genome is used to select additional files containing small RNA and RefSeq gene annotations.

*Parameters:* None

*Output:* The output of this stage is a FASTA file containing the genomic windows surrounding exact matches.

*Required tools:* bedtools v2.17.0

*Motivation & description:* miRNA modifications include (1) RNA edits and (2) the addition of non-templated nucleotides to the 3’-end of the sequence. We make the simplifying assumption that every expressed miRNA will have at least one read that will exactly map to the genome (no edits, or additions). To capture miRNA related reads, we define regions surrounding exactly mapped reads as a set of genomic windows. Windows separated by fewer than 65 nucleotides are merged as they may represent miRNA precursor loci.

Merged windows are further extended by 5 nt on either end to capture any non-templated additions. Finally these windows are converted to FASTA format.

**Stage 4**: Align remaining reads to window library (mismatches allowed).

*Input:*

1. Unaligned reads (fileName.noHits).
2. Genomic window library (generated in stage 3).

*Parameters:*

1. Read quality offset (33/64): this is the offset added to the phred score in the FASTQ quality scores.
2. Genome [hsa, mmu] used to select annotation files for tRNAs, miRNAs, and other gene annotations.

*Output:* window.results files. A file is generated for each genomic window containing the details of the alignment including mismatch string and the fractional weight of the alignment. Each read is weighted by 1/N, where N is the number of places the read aligns with the same score.

*Required tools:* SHRiMP 2.2.2

*Motivation:* Our final alignment stage attempts to align any previously unaligned reads to the newly generated FASTA library of genomic windows (with mismatches allowed) using SHRiMP2. Specifically, the set of all possible “alignment seeds” containing one mismatch in the body and up to three mismatches at the 3’-end (depending on read length) is generated and used to align all reads to the genomic windows.

**
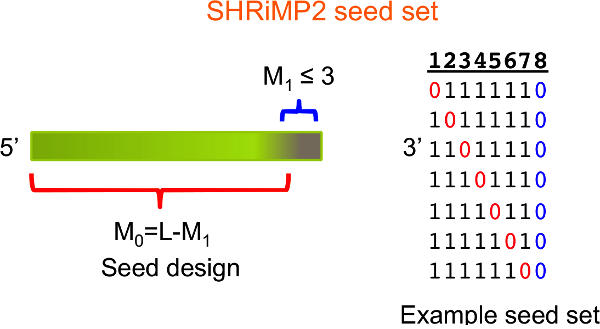
**

The number of mismatches allowed at the 3’-end (M_1_) for a read of length L is defined as: M_1_=0 if L<16, M_1_=1 if 16≤L<19, M_1_=2 if 19≤L≤23, and M_1_=3 if L>23. Finally, alignments are scanned and only the best alignments are retained. All reads mapping equally well to multiple loci are proportionally assigned to those loci.

**Stage 5:** Consolidation of results

*Input:* window.results files (output of Stage 4).

*Parameters:* Genome [hsa, mmu] used to select miRNA and other annotation files.

*Output:* TAB_3p_summary.txt, TAB_ed_summary.txt, TAB_lenDist_summary.txt: files detailing the reads 3’NTAs, edits and length variability (respectively).

*Required tools:* In-house Perl scripts

*Motivation:* By default aligned reads are grouped by 5’-start position (5’-isomiRs) and are annotated with respect to the 5’-start position of the reference (miRBase r18) miRNA at the same locus. For each 5’-isomiR, all reads with mismatches at the 3’-end are counted as 3’-NTAs. Mismatches within a read are counted as potential RNA edits.

**Candidate miRNA regulatory hub identification pipeline:**

This pipeline is intended to compute the predicted impact for each miRNA in a set (typically those highly expressed in a particular cell type) on a network of genes (typically those relevant to the study of a disease relevant to that cell type), and to predict candidate miRNAs that may act as regulatory hubs in that cell type for a particular disease or pathway.

*Inputs:*

1. A list of predicted target sites for miRNA families in the multiple-sequence aligned 3’-UTRs of all genes for a set of species. This output can be generated from TargetScan (<http://www.targetscan.org>).
2. A list containing the conservation (number of species) of each of the miRNA families included in the above TargetScan predictions. This list was parsed out of the TargetScan input files.
3. A list of high confidence protein-protein interactions. This list was derived from the STRING 9.0 database (http://string-db.org/) using only those interactions having an interaction score greater than 700 (high-confidence). All protein identifiers were mapped to their corresponding gene symbol, and this symbol must match those used in the TargetScan predictions.
4. A list of miRNA families: This list contains the miRNAs the user wishes to include in the simulations. All miRNAs must be in the TargetScan miRNA family name format, and must match the family names in the Target Scan output file.
5. Gene list: A list of genes to use as central nodes in a gene network. Typically this list includes a set of genes relevant to the study of a particular disease or pathway. Currently all gene names must match the gene symbol used in the TargetScan 3’-UTR sequence files.

*Parameters:*

1. ***C***: Conservation level. Requested minimum level of conservation of each miRNA and target site required for a miRNA - gene interaction to be scored in the simulation.
2. ***N***: Number of iterations. Requested number of random gene networks of similar design that are used to generate score distributions.
3. ***α***: Hub weighting. This parameter is used to weight the contribution of the number of high-confidence protein-protein interactions to the target scoring function.

*miRNA scoring algorithm:*

A gene network is compiled using the input files for (1) the input gene list and (2) each of the ***N*** requested random gene networks. The input gene network contains all genes in the input gene list that have a 3’-UTR listed in the target prediction files, a weighted set of scores for each target site within each gene, and the number of high confidence protein - protein interactions listed for that gene in the STRING 9.0 database. Each random gene network is generated by selecting a set of random genes having connectivity similar to each of those in the input gene list. A gene is said to have similar connectivity if the gene has a similar number of high confidence interactions in the STRING 9.0 database. To compute groups of genes with similar connectivity, we group each gene in the STRING 9.0 database by the number of high confidence protein-protein interactions that gene has. If any group contains fewer than 20 genes, the group is expanded to include neighboring groups (with both higher and lower number of interactions) until the new super-group contains at least 20 genes. Finally a score is computed for each gene network (input gene network and ***N*** random gene networks) each miRNA family in the input list, and an empirical p-value is computed. The p-value is calculated as ***p=(N_r_+1)/(N+1)***, where ***N_r_*** is the number of random gene networks in which the targeting score for a particular miRNA was greater or equal to the score of that miRNA in the input gene network. The miRNA targeting score is calculated using the following procedure:

For a gene network ***G(L,D,U)****:* where ***L*** is the list of genes in the network, ***D*** is the number of high confidence protein-protein interactions that each gene has, and ***U*** is the ratio of the average 3’-UTR length in the input gene network over the average 3’-UTR length in the current gene network (note: this value is one when scoring the input gene network).

foreach ***miR_i_*** in ***miRlist***  having a conservation of at least ***C***:

foreach ***gene_j_*** in ***L***:

foreach target site ***k*** of ***miR_i_*** in ***gene_j_***:

${ScoreA}_{ijk}=\left\{ \begin{matrix} 1.5 if 8mer-1a \\ 1.25 if 7mer-m8 \\ 1 otherwise \end{matrix} \right\}$

$${ScoreB}_{ijk}=\left\{ \begin{matrix} {ScoreA}_{ijk} & if site is consered in\geq\boldsymbol{C}\mathrm{species} \\ 0 & otherwise \end{matrix} \right\}$$

foreach ***miR_i_*** in ***miRlist***  having a conservation of at least ***C***:

$${Score}_{i}=0$$

foreach ***gene_j_*** in ***L***:

${ScoreC}_{ij}={ScoreB}_{ij0}$

foreach additional target site ***k*** of ***miR_i_*** in ***gene_j_***:

$${ScoreC}_{ij}+=\left\{ \begin{matrix} 0.5*{ScoreB}_{ijk} & if {(Pos}_{k}-{Pos}_{k-1})\leq8 \\ 1.5*{ScoreB}_{ijk} & if 8<{(Pos}_{k}-{Pos}_{k-1})<60 \\ {ScoreB}_{ijk} & if {(Pos}_{k}-{Pos}_{k-1})\geq60 \end{matrix} \right\}$$

where *Pos_k_* is the position of target site ***k*** within the 3’-UTR of ***gene_j_***.

$${Score}_{i}+=\boldsymbol{U}*{ScoreC}_{ij}\left( 1+\boldsymbol{\alpha}*log10\left( \boldsymbol{D}_{j} \right) \right)$$

${Score}_{i}={{Score}_{i}}/{size(\boldsymbol{L})}$
